# Activation of Endocannabinoid Receptor 2 as a Mechanism of Propofol Pretreatment-Induced Cardioprotection against Ischemia-Reperfusion Injury in Rats

**DOI:** 10.1155/2017/2186383

**Published:** 2017-07-26

**Authors:** Hai-Jing Sun, Yan Lu, Hao-Wei Wang, Hao Zhang, Shuang-Ran Wang, Wen-Yun Xu, Hai-Long Fu, Xue-Ya Yao, Feng Yang, Hong-Bin Yuan

**Affiliations:** ^1^Department of Anesthesiology, Changzheng Hospital Affiliated to Second Military Medical University, No. 415 Fengyang Road, Shanghai 200003, China; ^2^Department of Neurology, PLA Rocket Force General Hospital, No. 16 Xinjiekouwai Street, Beijing 100088, China; ^3^Department of Anesthesiology, PLA Rocket Force General Hospital, No. 16 Xinjiekouwai Street, Beijing 100088, China; ^4^Nursing School of Shanghai Jiguang Polytechnic College, No. 2859 Shuichan Road, Shanghai 201901, China; ^5^Hebei North University School of Medicine, Zhangjiakou, Hebei 075000, China; ^6^School of Pharmacy, Second Military Medical University, No. 325 Guohe Road, Shanghai 200433, China

## Abstract

Propofol pretreatment before reperfusion, or propofol conditioning, has been shown to be cardioprotective, while its mechanism is unclear. The current study investigated the roles of endocannabinoid signaling in propofol cardioprotection in an in vivo model of myocardial ischemia/reperfusion (I/R) injury and in in vitro primary cardiomyocyte hypoxia/reoxygenation (H/R) injury. The results showed that propofol conditioning increased both serum and cell culture media concentrations of endocannabinoids including anandamide (AEA) and 2-arachidonoylglycerol (2-AG) detected by LC-MS/MS. The reductions of myocardial infarct size in vivo and cardiomyocyte apoptosis and death in vitro were accompanied with attenuations of oxidative injuries manifested as decreased reactive oxygen species (ROS), malonaldehyde (MDA), and MPO (myeloperoxidase) and increased superoxide dismutase (SOD) production. These effects were mimicked by either URB597, a selective endocannabinoids degradation inhibitor, or VDM11, a selective endocannabinoids reuptake inhibitor. In vivo study further validated that the cardioprotective and antioxidative effects of propofol were reversed by selective CB2 receptor antagonist AM630 but not CB1 receptor antagonist AM251. We concluded that enhancing endogenous endocannabinoid release and subsequent activation of CB2 receptor signaling represent a major mechanism whereby propofol conditioning confers antioxidative and cardioprotective effects against myocardial I/R injury.

## 1. Introduction

Myocardial ischemia is the mostly seen cardiovascular complications during or after major surgeries with an incidence ranging from 1% to 7% [1, 2]. It is also the leading cause of perioperative morbidity and death [3]. With the introduction of immediate revascularization, reducing ischemia/reperfusion (I/R) injury is becoming a major obstacle for better recovery [4]. Ischemic conditioning, especially preconditioning, has been proved as a powerful strategy for mitigating myocardial I/R injury [5]. However, its clinical application was limited due to invasive procedures and the need to predict ischemia onset [6]. In this context, pharmacological intervention used before reperfusion is gaining attention [7].

As a widely used anesthetic, propofol is found to be cardioprotective in both experimental settings and clinical studies [8, 9]. The potential molecular mechanisms include antioxidation, anti-inflammation, or activating cardioprotective signaling pathways [10–12]. However, little is known about the direct upstreaming target or initiating molecule.

The endocannabinoid system comprises endocannabinoids, receptors (mainly CB1R and CB2R), and synthetic and degradative pathways [13]. Dozens of studies suggest that cardiovascular endocannabinoids play a role in myocardial I/R injury. Endocannabinoid release is enhanced after mouse myocardial I/R injury [14]. In human, increased plasma anandamide (AEA) and 2-arachidonoylglycerol (2-AG) levels were found in obese patients and were related with coronary circulatory dysfunction [15]. Both CB1R and CB2R signaling modulate remote ischemic preconditioning-induced cardioprotection [16–18]. Interestingly, propofol acts on endocannabinoid signaling. Patel et al. reported that propofol was a competitive inhibitor of fatty acid amide hydrolase (FAAH), which catalyzes the degradation of endocannabinoids with an IC50 of 52 *μ*M [19]. Propofol used at clinical dosage could significantly elevate brain endocannabinoid levels. Moreover, the sedative, amnesic, and analgesic effects of propofol could be modulated by endocannabinoid signaling [19–21]. Taken together, these results motivated us to hypothesize that propofol might act through endocannabinoid system to protect the myocardium against I/R injury.

To testify this hypothesis, we first examined the effects of propofol on endocannabinoid release in in vivo and in vitro. Then by modulating endocannabinoid levels, the relationship between propofol-induced cardiomyocyte protection and endocannabinoid release was investigated in in vivo rat cardiac I/R injuries and in vitro cardiac H/R injury models. Moreover, we demonstrated that propofol-induced myocardial protection was dependent on CB2R signaling.

## 2. Materials and Methods

### 2.1. Animals

All procedures concerning animal use had got the approval by the Animal Care and Use Committee of the Second Military Medical University (SMMU, Shanghai, China) and were confirmed to the Guide for the Care and Use of Laboratory Animals from the US National Institutes of Health (NIH Publication number 85-23, revised 1996). Newborn (P1–3) Sprague-Dawley rats (SIPPR/BK, Shanghai, China) were used for cardiac cell harvest and culture. 12–16-week-old young male rats (250–350 g) were kept for the in vivo myocardial I/R injury experiment. The animals were housed in a temperature-controlled (22 ± 2°C) room under a 12 : 12 hour light-dark cycle (lights on at 8:00 am) with ad libitum access to food and water.

### 2.2. Primary Cardiomyocyte Culture, In Vitro H/R Injury Model, and Propofol Conditioning

The methods for cell culture and H/R model were similar to our previous publications. Briefly, ventricles harvested from newborn rats were subjected to 15 min digestion with 0.1% trypsin (Gibco, New York, USA). After centrifugation, the remaining was dissociated using 0.1% collagenase II (Gibco) and incubated at 37°C for 3 hours. Then, cells were separated by filtering and further centrifugation. The collected cells were resuspended in NCS-DMEM with penicillin and streptomycin and cultured for 1 hour in a thermotank to drop fibroblasts. The purified cells were then cultured at 37°C, and 0.1 mM of bromodeoxyuridine (Sigma, St. Louis, MO, USA) was added during the first two days to inhibit fibroblast growth.

Before hypoxia, the culture medium was changed to low-glucose (5.56 mM) and serum-free DMEM. Hypoxia was achieved using a hypoxic incubator (Thermo Forma Anaerobic System Model 1025; Marietta, Ohio, USA) with 5% CO_2_ and 95% N_2_ for 12 hours. After hypoxia, the medium was switched to the maintenance medium under normal oxygen concentration for up to 4 hours of posthypoxic reoxygenation. Propofol conditioning was performed by incubating cardiomyocytes with 50 *μ*M of propofol 1 hour prior to hypoxia until the end of hypoxia [12]. The concentration was reported to fall in the range of narcotic concentrations in humans [22] and was about IC50 of propofol on mouse brain cell membrane FAAH activity [19].

### 2.3. Rat In Vivo Myocardial I/R Injury Model and Propofol Conditioning

The young rats were anesthetized using pentobarbital sodium (40 mg/kg) and ventilated on a rodent respirator. After thoracotomy at the fourth intercostal space, the left anterior descending coronary artery was either manipulated or occluded using a reversible coronary artery snare. I/R injury was performed by tightening the snare for 30 minutes (ischemia) and then loosening it for reperfusion for a given time period [23]. Specifically, peripheral blood samples were collected at 0, 1, 2, and 4 hours after ischemia for endocannabinoid measuring and at 2 hours after ischemia for cTnI detection and at 24 hours for determining MDA, MPO, and SOD levels. Infarction area was confirmed after 24-hour reperfusion. Propofol (Sigma, resolved in 20% intralipid) conditioning was performed by a bolus intravenous injection of 10 mg/kg of propofol followed by continuous infusion at a rate of 39 mg/kg·h from one hour before ischemia until the end of ischemia [24, 25]. Propofol at this rate of infusion was found to be cardioprotective [24] and maintains deep sedation with a mean arterial plasma propofol concentration of 4.4 mg/L (25 *μ*M) [26].

### 2.4. Experimental Design

The study aimed to test the following hypotheses. Firstly, propofol could modulate cardiac endocannabinoid signaling which comprised endocannabinoid release, receptor expression, and downstreaming signaling activation. Secondly, manual control of endocannabinoid release had an effect on propofol conditioning-induced cardioprotection. Thirdly, CB2R receptor but not CB1 receptor signaling was responsible for propofol-induced cardioprotection.

To achieve these goals, the following sets of experiments were conducted.

#### 2.4.1. Experiment 1: Effects of Propofol Conditioning on Cardiac Endocannabinoid Signaling In Vitro and In Vivo

The cardiomyocytes (in vitro) or rats (in vivo) were divided into four groups: control (sham), propofol, H/R (I/R), and propofol + H/R (I/R). H/R was achieved by hypoxia for 12 hours followed by up to 4-hour reoxygenation. 10 minutes after the beginining of propofol exposure and 0, 1, 2, and 4 hours after reoxygenation or reperfusion the cultured cell medium or peripheral blood was collected for endocannabinoid determination. The cultured cells were also harvested for measuring mRNA and protein levels of CB1R and CB2R at 4 hours of reoxygenation.

#### 2.4.2. Experiment 2: Effects of Endocannabinoid Release on Propofol Conditioning-Induced Cardiomyocyte Protection from H/R

Neonatal rat ventricle cells were subjected to H/R with or without propofol conditioning. Selective FAAH inhibitor URB597 and endocannabinoid reuptake inhibitor VDM11 were further used to increase endocannabinoid levels before propofol conditioning. URB597 was purchased from Cayman Chemicals (Ann Arbor, MI, USA) and diluted to a concentration of 1 *μ*M before use. The timing of URB597 incubation was 1.5 hours before hypoxia until the end of propofol conditioning [27, 28]. VDM11 (Cayman) was used at a final concentration of 10 *μ*M. The time to start exposure and duration was the same with URB597 [29]. The following indicators were detected at 4 hours of reoxygenation: cell viability, cell apoptosis, culture medium-lactated hydrogenase (LDH), and MDA and SOD concentrations as well as cell ROS production.

#### 2.4.3. Experiment 3: Effects of CB1R and CB2R Signaling in Propofol-Induced Cardioprotection against I/R Injury

Young rat myocardial I/R injury models were used. Before ischemia, the rats were preconditioned with or without propofol. We further tested the role of receptor signaling in propofol conditioning by intravenous injection of selective CB1R antagonists AM251 (Sigma, 1 mg/kg) or selective CB2R antagonists AM630 (Sigma, 1 mg/kg) 1.5 hours prior to ischemia irrespective of propofol conditioning or not. The dosage and timing of AM251 or AM630 were taken from the study by Hajrasouliha et al. [17]. The two drugs were used separately in our study which comprised seven groups with six rats for each group. 24 hours after reperfusion, infarct area was evaluated using Evans blue plus TTC staining. Serum cTnI levels were measured 2 hours after ischemia. Oxidative-redox state indicators including serum MDA, MPO, and SOD were also measured at 24 hours of reperfusion.

### 2.5. LC-MS/MS Determination of Endocannabinoid Concentrations

Peripheral blood samples and cell culture media samples were collected and stored immediately at −80°C until extraction to reduce degradation [30]. For sample preparation, 100 *μ*L of plasma or culture fluid was first precipitated with ice-cold methanol/Tris buffer (50 mM, pH 8.0) and centrifuged at 12500 rpm for 10 min (4°C) to remove the precipitates. The supernatants were then extracted with ice-cold CHCl3: MeOH (2 : 1) and washed with CHCl3. The extracts were dried by nitrogen flow and stored at −80°C. Before measurement, the analytes were reconstituted with MeOH.

Concentrations of AEA and 2-AG were detected by stable isotope liquid chromatography/tandem mass spectrometry (LC-MS/MS) at the School of Pharmacy, Second Military Medical University, using the method adapted from Sergi et al. [31]. The method has been validated previously [32]. Precision (RSD%) was always smaller than 15%, and accuracy ranged from 85% to 115%. Ion suppression due to matrix components was not significant for both AEA (0.90) and 2-AG (0.92). The limits of detection for AEA and 2-AG were 0.2 and 0.7 pmol/mL, respectively.

Briefly, the Waters ACQUITY UPLC Console UHPLC system (Milford, MA, USA) was coupled to a TSQ Quantum triple Quad mass spectrometer with a TurboIonSpray (ESI) source (Thermo, Waltham, MA, USA). Liquid chromatographic separation was achieved by Athena C18-WP 50 × 2.1 mm (3 *μ*m, Waters). The mobile phases included water (phase A) and 0.1% formic acid in methanol (phase B) with a flow rate of 0.3 mL/min. Gradient elution was flowed at and was held at 10% B for the first 2.5 min, followed by 100% B at 2.5 min, 90% B at 3 min, 100% B at 4 min, and 60% B from 4.5 to 5 min. The injected volume was 10 *μ*L and the column temperature was 25°C. AEA and 2-AG were detected in a positive ion mode using electron spray ionization and selective reaction monitoring mode of acquisition. The ion spray voltage was set at 3500 V. The ion pairs for the detection of AEA and 2-AG were m/z: 348.2 → 62.00 and m/z: 379.2 → 287.2, respectively. For each sample, levels of AEA and 2-AG were measured in duplicates against standard curves and were expressed as pmol/mL.

### 2.6. FAAH Activity Assay

FAAH activity was determined using a commercial FAAH Inhibitor Screening Assay Kit (Cayman Chemicals, Ann Arbor, MI, USA). The kit used a fluorescence based to screening FAAH inhibitors. 7-amino-4-methylcoumarin (AMC) arachidonoyl amide was used as the substrate for human recombinant FAAH, and the products AMC were detected using an excitation wavelength of 340–360 nm and an emission wavelength of 450–465 nm. The effects of 0.1, 1, 10, 50, 100, and 200 *μ*M of propofol on FAAH activity were tested.

### 2.7. Flow Cytometry Measuring of Cell Apoptosis

Cell apoptosis was detected using Annexin-V/PI double-staining method under flow cytometry [12]. The Annexin-V apoptosis detection kit APC (eBioscience, San Diego, CA, USA) was used. Briefly, after washing with ice-cold PBS and resuspension with binding buffer, the suspension was mixed with 5 *μ*L of APC and 5 *μ*L of PI and then loaded on to a FACSCalibur flow cytometer (BD; San Jose, CA, USA) for detection.

### 2.8. Flow Cytometry Measuring of Intracellular ROS Levels

ROS was measured using the fluorescent probe CM-H2DCFDA (Invitrogen; Carlsbad, CA, USA). An excitation wavelength of 488 nm and emission wavelength of 525 nm were used for flow cytometry measuring of ROS. The mean fluorescence intensity (MFI) was used to indicate levels of ROS.

### 2.9. Cell Viability Analysis

Cardiomyocyte viability was detected using a commercial CKK-8 kit (Beyotime, Haimen, China). Briefly, the cells were seeded in 96-well cell culture at 1 × 10^4^ cells/well. After reoxygenation, 100 *μ*L of 10% CKK-8 solution was added and incubated for 2 hours. The plates were shifted to a microplate reader for absorbance recording at 450 nm.

### 2.10. LDH, MDA, and SOD Concentrations

A LDH assay kit (Beyotime), a MDA assay kit (Beyotime), and a SOD kit (Kumamoto, Japan) were used for the detection of cell culture media and serum concentrations. The manufacturer's instructions were followed. LDH, MDA, and SOD concentrations were calculated based on the absorbance value at 450 nm, 530 nm, and 560 nm, respectively.

### 2.11. ELISA Measuring of Serum Cardiac Troponin I (cTnI) and MPO Concentrations

The commercial kits from Cell Signaling Technology (Danvers, MA, USA) were used for detecting concentrations of serum cTnI and MPO. Procedures were performed according to the manufacturer's instructions. Each sample was detected at twice and the mean concentration was used.

### 2.12. Real-Time PCR for CB1R, CB2R, and FAAH mRNA Levels of Detection

Cardiomyocytes were harvested after treatment, and RNA was isolated using a commercial Total RNA Kit (Feijie, Shanghai, China). The concentrations of RNA were measured with a microplate spectrophotometer (Bio-Rad, Hercules, CA, USA) at 260 nm. 2 *μ*g of total RNA was used for cDNA synthesis with the reverse transcriptase kit (Takara, Japan) according to the manufacturer's instructions. Amplification was performed with the following parameters: denaturation at 95°C for 2 min, followed by 40 cycles of denaturation at 95°C for 10 s, annealing at 63°C for 15 s, and extension at 72°C for 30 s. *β*-actin was used as an internal control. Sequences of primers used were as follows: CB1R forward: 5′-CAGAAAATGCACGATGAGGA-3′; CB1R reverse: 5-′GTACAGCGATGGCAGCTGCTG-3′; CB2R forward: 5′-CCGCTCATGGGGTGGACTTG-3′; CB2R reverse: 5′-CCGCAGGGCATAAATG ATAGGAT-3′; FAAH forward: 5′-TGGAGCGAGTTGTGGATTGTT-3′; FAAH reverse: 5′-AGGGG′IAGTGATGTCCAGGAAGTA-3′; *β*-actin forward: 5′-GG GAAATCGT GCGTGACATT-3′; and *β*-actin reverse: 5′-CGGATGTCAACGTCACACTT-3′. The relative expression levels of CB1R and CB2R (normalized to that of *β*-actin) were determined using 2^-ΔΔCt^ method.

### 2.13. Western Blot for Detection of CB1R, CB2R, and FAAH1 Protein Concentrations

Cardiomyocytes were lysed in ice-cold RIPA lysis buffer with 1% protease cocktail. Protein concentrations were then determined using the bicinchoninic acid (BCA) protein assay kit (Beyotime). 40 *μ*g of protein was loaded and separated in 10% SDS-polyacrylamide gel. Then, the protein was transferred to a nitrocellulose membrane for detection. The membranes were immersed overnight in the corresponding primary antibodies at 4°C. The CB1R and FAAH antibody was purchased from Abcam (UK) and CB2R antibody from Millipore (Billerica, MA, USA). Both were diluted at 1 : 1000. After rinsing, the membranes were incubated with a horseradish peroxidase-conjugated secondary antibody for 1 hour at room temperature. The blots were visualized by an enhanced chemiluminescence reaction (ECL) system and photographed by ChemiDoc™ XRS+ System (Bio-Rad). GAPDH (1 : 5000; CST) was used as an internal control. Band densitometry analysis was performed using Quantity One software (Bio-Rad).

### 2.14. Determination of Infarct Area by Triphenyltetrazolium Chloride (TTC) Staining

Infarct size was examined 24 hours after ischemia using the TTC staining method [33]. Briefly, the hearts were first perfused with saline to remove blood. The left descending artery was then again ligated, and 1% Evans blue was used to perfuse the nonischemic parts of the heart. After rinsing, the heart ventricles were sectioned and incubated in 1% TTC for 20 minutes to stain the viable myocardium brick red. The samples were then fixed in 10% formalin, and the slices were photographed. The infarcted area (unstained by TTC) and ischemic risk area (unstained by Evans blue) were measured using Image-Pro Express software (Olympus, Japan). Infarct size was expressed as a percentage of the ischemic risk area.

### 2.15. Statistical Analysis

Statistics were performed using SPSS 19.0 (IBM, Armonk, NY, USA). Data were expressed as mean ± SD (standard deviation). Normal distribution was tested before all analysis. Two-way analysis of variance (ANOVA) for repeated measures followed by post hoc Bonferroni tests was used to evaluate dynamic changes of endocannabinoid concentrations across time and group. One-way ANOVA and post hoc Bonferroni tests (homogeneity of variance assumed) or Games-Howell tests (homogeneity of variance not assumed) were used to analyze differences among groups in the infarct area, CB1R and CB2R mRNA and protein levels, cell apoptosis, cell viability, LDH leak, SOD activities, ROS production, and cTnI, MDA, and MPO concentrations. For all comparisons, a value of *P* < 0.05 (two tailed) was considered statistically significant.

## 3. Results

### 3.1. Propofol Conditioning Enhanced Cardiac Endocannabinoid Release In Vivo

**Figure 1:**
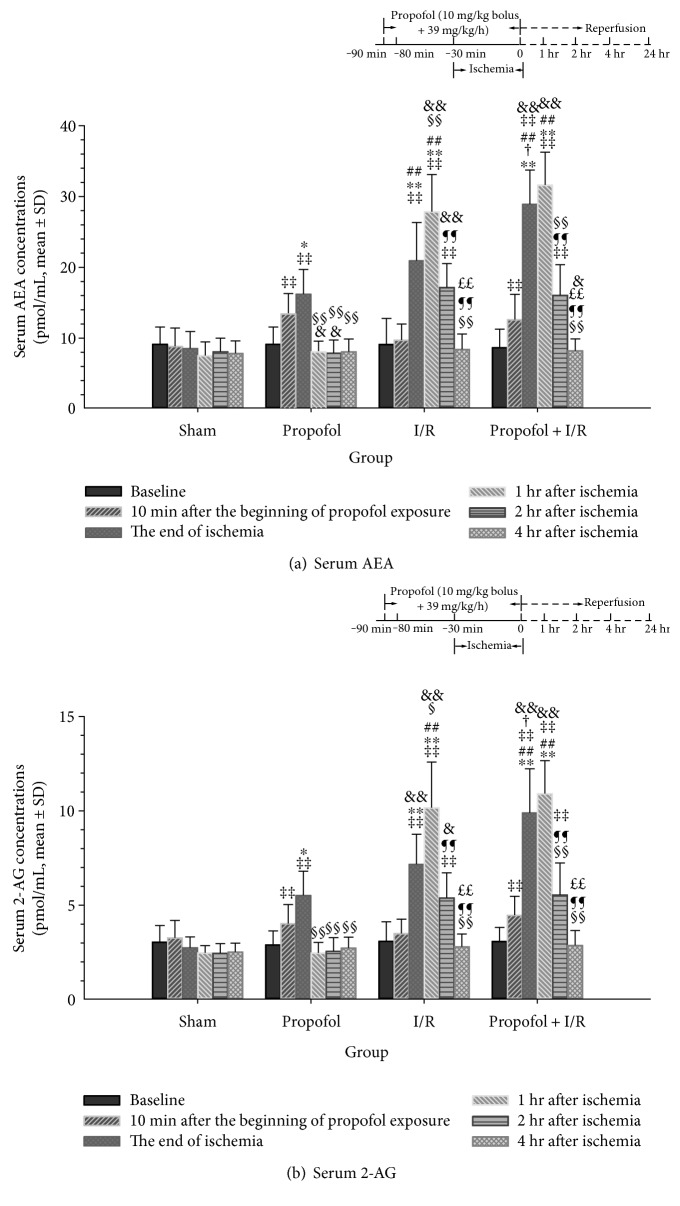
Effects of propofol conditioning on endocannabinoid release in vivo. (a) Serum AEA concentrations among groups. (b) Serum 2-AG concentrations among groups. In the rat myocardial ischemia/reperfusion (I/R) injury model, propofol conditioning was achieved by an intravenous bolus of 10 mg/kg followed by continuous infusion at a rate of 39 mg/kg·h from one hour before ischemia until the end of ischemia. Peripheral blood was collected at 10 minutes after the beginning of propofol conditioning and at 0, 1, 2, and 4 hours after ischemia. Endocannabinoids including AEA and 2-AG were detected by LC/LC-MS. The results showed that propofol conditioning and I/R enhanced cardiac endocannabinoid release in vivo. *N* = 6 per group for each time point. ∗: *P* < 0.05; ∗∗: *P* < 0.01 versus sham. *P* < 0.05; ##: *P* < 0.01 versus propofol. †: *P* < 0.05 versus I/R. *P* < 0.05; ‡‡: *P* < 0.01 versus baseline. &: *P* < 0.05; &&: *P* < 0.01 versus 10 mins after the beginning of propofol exposure. §: *P* < 0.05; §§: *P* < 0.01 versus the end of ischemia. *P* < 0.05; ¶¶: *P* < 0.01 versus 1 hr after ischemia. *P* < 0.05; ££: *P* < 0.01 versus 2 hr after ischemia. I/R, ischemia/reperfusion.

In the myocardial I/R model, we first assessed the changes of serum AEA and 2-AG after ischemia and propofol conditioning using LC-MS/MS. Two-way ANOVA with repeated measures analysis identified significant time-dependent (*P* < 0.001) and group-dependent (*P* < 0.001) effects on serum AEA concentations. Post hoc Bonferroni tests found that I/R (*P* < 0.001) and propofol conditioning with I/R (*P* < 0.001) increased serum AEA concentrations as depicted in Figure 1(a). Serum AEA concentrations were similar at baseline among four groups. I/R significantly increased AEA levels at the end of ischemia (95% confidence interval for difference (CI-D), 8.23–15.42 pmol/mL), 1 hour (95% CI-D, 14.30–23.21 pmol/mL) and 2 hours after ischemia (95% CI-D, 3.29–12.80 pmol/mL). Propofol alone increased serum AEA levels at 10 minutes after the beginning of exposure (95% CI-D, 1.81–5.63 pmol/mL) and at the time point corresponding to end of ischemia (95% CI-D, 2.90–10.09 pmol/mL), but not at other time points. Under conditions of I/R, propofol conditioning induced significant increases in AEA concentrations both at the end of ischemia and at 1 and 2 hours during postischemic reperfusion (95% CI-D, 16.66–23.85, 18.50–27.42, and 2.64–12.15 pmol/mL, resp.). A higher AEA level was observed at the end of ischemia in propofol conditioning group compared with I/R alone (95% CI-D, 0.91–15.06 pmol/mL) and propofol alone (95% CI-D, 5.63–19.78 pmol/mL, Figure 1(a)).

A similar time- and group-dependent change in serum 2-AG concentration was observed. As shown in Figure 1(b), propofol alone increased serum 2-AG levels at 10 minutes after the beginning of exposure (95% CID, 0.28–1.98 pmol/mL (propofol group) and 0.54–2.24 pmol/mL (propfol + I/R group)). Propofol alone (95% CID, 0.11–5.43 pmol/mL, *P* = 0.038), I/R alone (95% CID, 1.76–7.08 pmol/mL, *P* = 0.001), and propofol preconditioning combined with I/R (95% CID, 4.45–9.80 pmol/mL, *P* < 0.001) increased 2-AG concentrations immediately after ischemia as compared to sham and rats receiving propofol conditioning showed the highest serum 2-AG concentrations. The increase in serum 2-AG concentrations was observed only in I/R (95% CID, 5.02–9.11 pmol/mL) and propofol + I/R (95% CID, 5.78–9.88 pmol/mL) groups at 2 hours during postischemic reperfusion compared with sham. No change was found for the four groups at 4 hours after ischemia compared with baseline (Figure 1(b)).

### 3.2. Propofol Conditioning Enhanced Cardiac Endocannabinoid Release In Vitro

**Figure 2:**
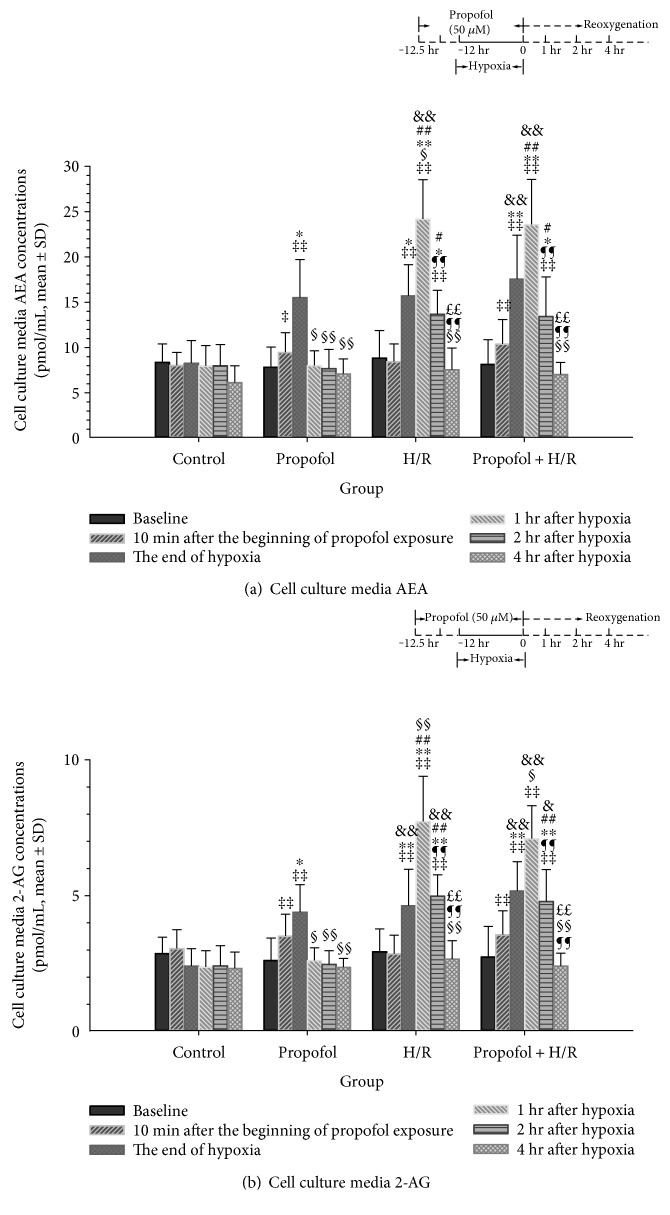
Effects of propofol conditioning on cardiomyocyte endocannabinoid release. (a) Cell culture media AEA concentrations among groups. (b) Cell culture media 2-AG concentrations among groups. In the cardiomyocyte hypoxia/reoxygenation (H/R) injury model, propofol conditioning was achieved by continuous incubation at a concentration of 50 *μ*M from 1 hour prior to hypoxia until the end of hypoxia. Cell culture media were collected at 10 minutes after the beginning of propofol conditioning and at 0, 1, 2, and 4 hours after hypoxia. Endocannabinoids including AEA and 2-AG were detected by LC/LC-MS. The results showed that propofol conditioning and I/R enhanced cardiac endocannabinoid release in vivo. *N* = 6 per group for each time point. ∗: *P* < 0.05; ∗∗: *P* < 0.01 versus control. #: *P* < 0.05; ##: *P* < 0.01 versus propofol. ‡: *P* < 0.05; ‡‡: *P* < 0.01 versus baseline. &: *P* < 0.05; &&: *P* < 0.01 versus 10 mins after the beginning of propofol exposure. §: *P* < 0.05; §§: *P* < 0.01 versus the end of hypoxia. *P* < 0.05; ¶¶: *P* < 0.01 versus 1 hr after hypoxia. *P* < 0.05; ££: *P* < 0.01 versus 2 hr after hypoxia. H/R, hypoxia/reoxygenation.

Cardiac endocannabinoids in vivo are a sum of endocannabinoids from peripheral circulation, local blood cells, mast cells, endothelia, or cardiomyocytes [34]. To detect the direct effects of propofol on cardiomyocyte AEA and 2-AG release, we then measured cell culture media endocannabinoid concentrations in the in vitro cardiomyocyte H/R injury model.  Figure 2() summarizes differences in cell culture media AEA (a) and 2-AG (b) levels as a function of different treatment groups at baseline, at 10 minutes after the beginning of propofol exposure, at the end of hypoxia, and at 1, 2, and 4 hours of posthypoxic reoxygenation.

AEA concentrations were similar at baseline among four groups. After 10 minute propofol incubation, cell culture media AEA concentrations were significantly increased in both propofol and propofol + H/R groups (95% CID, 0.04–3.22 and 0.66–3.84 pmol/mL, resp.). At the end of hypoxia or the time points corresponding to end of hypoxia in the control and propofol groups, significant increases of AEA concentrations were found in the propofol (95% CID, 3.02–12.34 pmol/mL), H/R (95% CID, 2.21–11.53 pmol/mL), and propofol preconditioning (95% CID, 4.75–14.08 pmol/mL) group but not the control group (95% CID, −4.76–4.57 pmol/mL) compared with those at baseline (Figure 2(a)). The increase of AEA by propofol alone was not found at 1, 2, and 4 hours of posthypoxic reoxygenation (all *P* = 1 versus baseline), suggesting a transient effect of propofol on cardiomyocyte AEA release. H/R itself increased AEA concentrations for more than 2 hours (95% CID, 2.21–11.53 pmol/mL at the end of hypoxia, 95% CID, 10.97–19.70 pmol/mL at 1 hour of reoxygenation, and 95% CID, 1.31–8.36 pmol/mL at 2 hours of posthypoxia). Propofol preconditioning combined with H/R also enhanced AEA release at 1 (95% CID, 11.07–19.79 pmol/mL, *P* < 0.001) and 2 hours (95% CID, 1.78–8.83 pmol/mL, *P* = 0.029) of reoxygenation compared with those at baseline (Figure 2(a)).

A similar time- and group-dependent change in 2-AG concentrations was observed. As shown in Figure 2(b), increased 2-AG concentrations were observed in propofol and propofol + H/R group at 0 minutes after the beginning of propofol exposure (95% CID, 0.33–1.47 and 0.25–1.39 pmol/mL, resp.). Propofol alone, H/R alone, and propofol preconditioning combined with H/R increased 2-AG concentrations immediately after hypoxia compared with those at baseline (95% CID, 0.72–2.83, 0.65–2.75, and 1.38–3.49 pmol/mL, resp.). The increased 2-AG concentration was observed only in H/R and propofol + H/R groups at 2 hours after hypoxia (95% CID, 0.81–3.30 and 0.80–3.29 pmol/mL, resp.). No significant difference was found for the four groups at 4 hours after hypoxia compared with baseline (all *P* = 1 versus baseline, Figure 2(b)).

### 3.3. Propofol Conditioning Increased Cardiomyocyte CB1R and CB2R mRNA and Protein Levels

**Figure 3:**
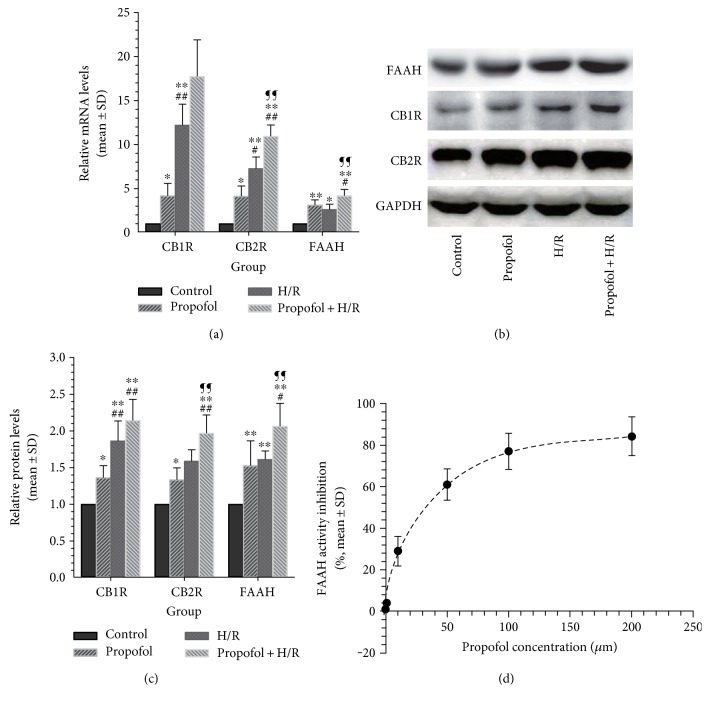
Effects of propofol conditioning on cardiomyocyte endocannabinoid receptors and FAAH transcription and expression in vitro (a–c) and effects of propofol on FAAH activity in vitro (d). (a) Propofol enhanced CB1R, CB2R, and FAAH mRNA transcription in cardiomyocytes. (b and c) Propofol increased cardiomyocyte CB1R, CB2R, and FAAH protein levels. (d) Propofol inhibited FAAH activity in a dose-dependent manner. *N* = 5 per group. ∗: *P* < 0.05; ∗∗: *P* < 0.01 versus control. #: *P* < 0.05; ##: *P* < 0.01 versus propofol. ¶¶: *P* < 0.01 versus H/R.

Apart from endocannabinoid release, the activity/activation of corresponding receptors in vitro was also investigated using real-time PCR and Western blot. As shown in Figure 3(a), post hoc Games-Howell tests revealed that both propofol conditioning (*P* = 0.025) and H/R (*P* = 0.002) increased CB1R mRNA transcription as compared with control. A similar increase in CB2R mRNA levels was also observed in propofol (*P* = 0.013) and H/R (*P* = 0.002) groups compared with control. A trend of increase in CB1R mRNA levels was found with no statistical significance (*P* = 0.009 versus control). Propofol combined with H/R also significantly enhanced CB1R (*P* = 0.003 versus control) and CB2R (*P* < 0.001 versus control) mRNA transcription. Compared with H/R, propofol exposure before hypoxia further increased (*P* = 0.009) CB2R mRNA levels.

With regard to protein levels of receptor expression, both propofol conditioning (*P* = 0.025) and H/R (*P* = 0.007) increased CB1R protein levels compared with control. CB2R protein translation was also enhanced by propofol conditioning and H/R (*P* = 0.034 and 0.044 versus control for propofol + H/R and H/R, resp.). Propofol combined with H/R also increased CB1R (*P* = 0.003) and CB2R (*P* = 0.003) protein levels compared with control (Figures 3(b) and 3(c)). Propofol + H/R further increased CB2R protein levels compared with H/R alone (*P* = 0.014). A trend of further increase in CB1R protein levels by propofol conditioning was found despite no statistical significance (*P* = 0.327 versus control).

### 3.4. Propofol Increased Cardiomyocyte FAAH mRNA and Protein Levels and Inhibits FAAH Activity In Vitro

As shown in Figure 3(), both propofol conditioning (*P* = 0.004) and H/R (*P* = 0.013) increased FAAH mRNA levels as compared with control (Figure 3()). A similar increase in FAAH protein levels was also observed in propofol (*P* = 0.003) and H/R (*P* = 0.001) groups compared with control. Propofol conditioning combined with H/R was associated with the most increased FAAH mRNA transcription and protein expression. The in vitro study found that propofol delivered in intralipid concentration dependently decreased the activity of FAAH with an IC50 of about 28 *μ*M (Figure 3(d)).

### 3.5. Propofol Conditioning-Induced Antihypoxic and Antioxidative Effects in Cardiomyocytes In Vitro Were Mimicked by Exogenous Increasing of Endocannabinoid Concentrations

To investigate the relationship between propofol-induced endocannabinoid release and cardiomyocyte protection against H/R injury, selective FAAH inhibitor URB597 and endocannabinoid reuptake inhibitor VDM11 were further applied to increase endocannabinoid levels in the absence or presence of propofol conditioning in cardiomyocytes subjected to H/R.

**Figure 4:**
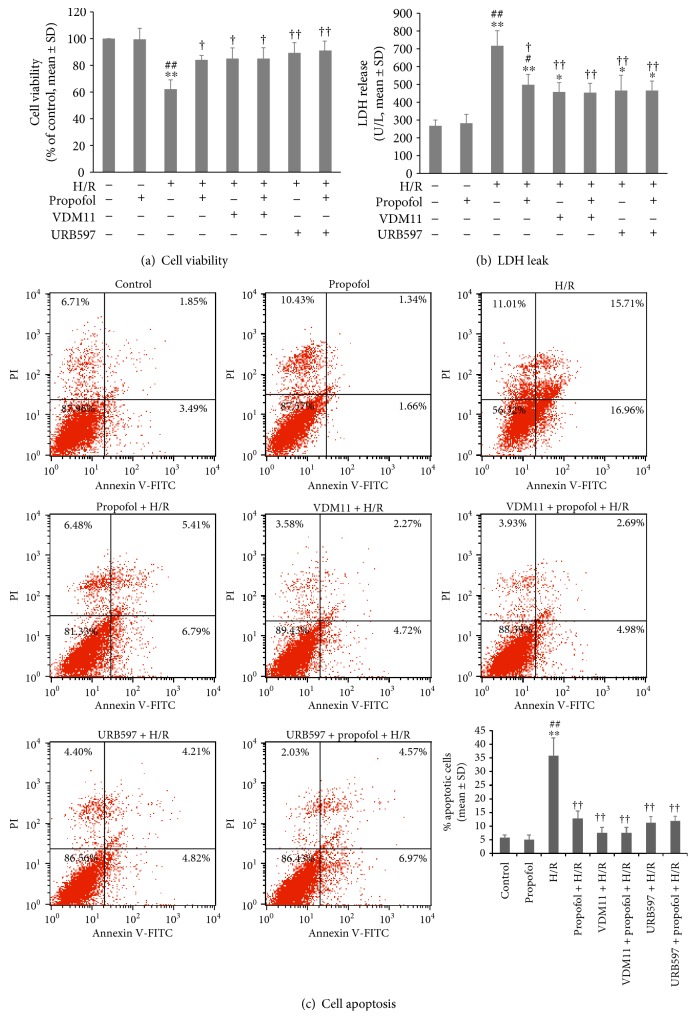
Effects of propofol conditioning, URB pretreatment, and VDM11 pretreatment on cell viability (a), LDH leak (b), and cell apoptosis (c) in vitro. URB597 (1 *μ*M) or VDM11 (10 *μ*M) was added 1.5 hours before hypoxia until the end of propofol conditioning. Cell viability was detected using the MTT kit. LDH release was measured using a LDH assay kit. Cell apoptosis was detected using Annexin-V/PI double-staining method under flow cytometry. The results showed that propofol conditioning-inhibited hypoxia/reoxygenation (H/R) induced decrease in cell viability (a), increase in cell LDH leak (b), and apoptosis (c). *N* = 6 per group. ∗: *P* < 0.05; ∗∗: *P* < 0.01 versus control. #: *P* < 0.05; ##: *P* < 0.01 versus propofol. †: *P* < 0.05; ††: *P* < 0.01 versus H/R.

As shown in Figure 4(a), propofol significantly increased cell viability against H/R (*P* = 0.029), and URB597 (*P* = 1 versus propofol + H/R) and VDM11 (*P* = 0.487 versus propofol + H/R) pretreatment showed similar cardioprotective effects to propofol conditioning. Moreover, no synergistic effect was found between propofol and URB597 (*P* = 1 versus propofol + H/R) or between propofol and VDM11 (*P* = 0.5 versus propofol + H/R) groups.

The posthypoxic LDH leak was shown in Figure 4(b), which demonstrated that propofol conditioning reduced cell LDH leak compared with H/R (*P* = 0.013). The LDH reducing effects were also seen in URB597 (*P* = 0.003) and VDM11 (*P* = 0.002) pretreatment groups. URB597 (*P* = 1) or VDM11 (*P* = 1) exposure before propofol conditioning did not enhance or suppress propofol-induced reduction in LDH leak.

The potential effects of URB and VDM11 pretreatment on propofol-induced inhibition of cell apoptosis were also evaluated. The flow cytometry results showed that similar to propofol conditioning, both URB and VDM11 pretreatment could inhibit H/R-induced cell apoptosis (*P* < 0.001, URB597 + H/R versus H/R; *P* < 0.001, VDM11 + H/R versus H/R). When propofol was used together with URB or VDM11, no further reduction in cell apoptosis rate was found (*P* = 1, propofol + H/R versus propofol + URB597 + H/R; *P* = 1, propofol + H/R versus propofol + VDM11 + H/R, Figure 4(c)).

**Figure 5:**
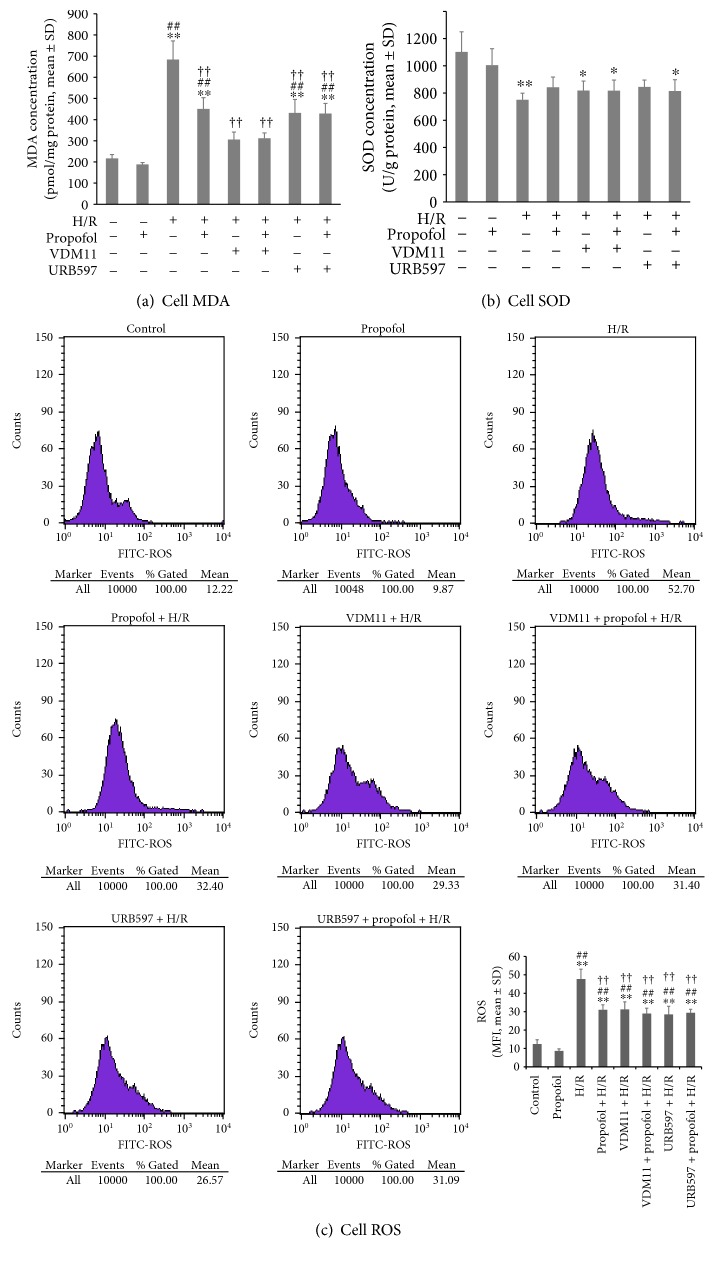
Effects of propofol conditioning, URB pretreatment, and VDM11 pretreatment on cardiomyocyte MDA concentrations (a), SOD concentrations (b), and ROS productions (c). In the in vitro cardiomyocyte hypoxia/reoxygenation (H/R) model, propofol (50 *μ*M) was incubated to achieve cardioprotection. Selective FAAH inhibitor URB597 and endocannabinoid reuptake inhibitor VDM11 were further used to increase endocannabinoid levels before propofol conditioning. The results showed that propofol conditioning was cardioprotective through decreasing oxidation (a and c). URB597 and VDM11 mimic the effects of propofol conditioning. The cardioprotective effects of propofol could not be exerted when URB597 or VDM11 was pretreated. *N* = 6 per group. ∗: *P* < 0.05; ∗∗: *P* < 0.01 versus control. ##: *P* < 0.01 versus propofol. ††: *P* < 0.01 versus H/R.

The cell viability, lactic acid dehydrogenase (LDH) leak, and cell apoptosis results above clearly suggested a similar pathway involved in endocannabinoid release and propofol conditioning-induced cardiomyocyte protection. We further tested the potential pathways in vitro. Propofol was an antioxidant [35], and previous studies suggested a role of inhibiting oxidative stress in propofol-induced protection against hypoxia or ischemia [11, 12, 36]. Our results found that compared to H/R, propofol conditioning could decrease cardiomyocyte malonaldehyde (MDA) concentrations (*P* = 0.001, Figure 5(a)) which was also seen in cardiomyocytes receiving VDM11 and URB597 incubation (both *P* < 0.001 versus HR). VDM11 combined with propofol conditioning did not further decrease MDA levels (*P* = 1 versus VDM11 + H/R; *P* = 0.093 versus propofol + H/R). Similarly, propofol conditioning after URB597 exposure did not further inhibit MDA production in cardiomyocytes (*P* = 1 versus URB597 + H/R).

Superoxide dismutase (SOD) is an endogenous antioxidase.  Figure 5(b) depicts cardiomyocyte SOD concentration changes after H/R with and without propofol, VDM11, and URB597 exposure. H/R alone significantly decreased SOD levels compared with control (*P* = 0.006). Propofol conditioning, VDM11 pretreatment, and URB597 pretreatment failed to increase SOD concentrations compared with H/R (all *P* = 1). An increase in SOD levels was found in neither VDM11 + propofol + H/R nor URB597 + propofol + H/R groups (both *P* = 1 versus H/R).

Reactive oxygen species (ROS) production changes among groups were further measured by flow cytometry, and the results were shown in Figure 5(c). H/R alone significantly increased ROS production compared with control (*P* < 0.001). The effects of H/R on ROS production were significantly inhibited by propofol conditioning, VDM11 pretreatment, and URB597 pretreatment (all *P* < 0.001 versus H/R). A further decrease in ROS levels was not seen when propofol conditioning was implemented after VDM11 or URB597 preexposure (both *P* = 1).

### 3.6. Propofol Conditioning-Induced Alleviation of Rat Myocardial Ischemia Reperfusion Injury and Antioxidation Were Reversed by Selective Antagonism of CB2R but Not CB1R

**Figure 6:**
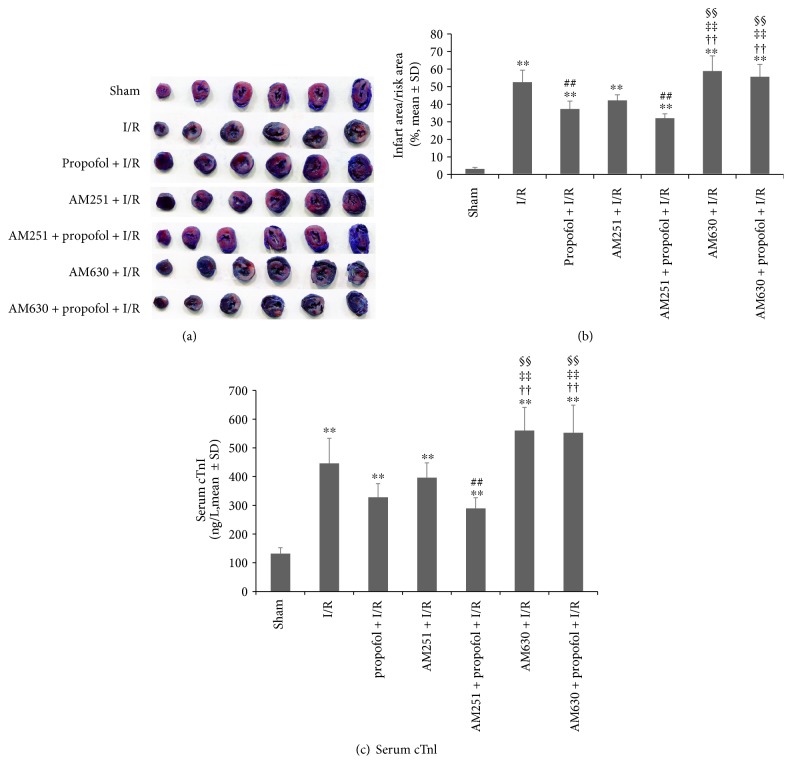
Effects of CB1R and CB2R signaling on propofol-induced cardioprotection in vivo. (a) Representative figures of cardiac ventricle slices showing myocardium infarct size among groups. (b) Histograms showing the relative infarct size among groups. Propofol was perfused from 1.5 hours before ischemia until the end of ischemia. Selective CB1R antagonist AM251 (1 mg/kg) or selective CB2R antagonist AM630 (1 mg/kg) was injected intravenously 30 minutes before propofol exposure. Infarct size was detected using TTC staining after 24-hour reperfusion. The results showed that propofol and AM251 pretreatment could reduce infarct size compared with pure ischemia/reperfusion. Pretreatment with AM630 fully antagonized the effects of propofol conditioning. (c) Histograms showing serum cTnI concentrations among groups. Propofol tended to reduce cardiac cTnI release at hours after ischemia, and selective CB2R antagonist AM630 (1 mg/kg) reversed the effect of propofol. *N* = 6 per group. ∗∗: *P* < 0.01 versus sham. ##: *P* < 0.01 versus I/R. ††: *P* < 0.01 versus propofol + I/R. ‡‡: *P* < 0.01 versus AM251 + I/R. §§: *P* < 0.01 versus AM251 + propofol + I/R.

We further confirmed the roles of endocannabinoid signaling, especially the roles of receptor activation (CB1R and CB2R) in propofol conditioning-induced cardioprotection in vivo. As shown in Figure 6, propofol conditioning significantly reduced rat heart infarct size compared with I/R (*P* = 0.001). The use of selective CB1R antagonist AM251 tended to be cardioprotective but difference did not reach statistical significance (*P* = 0.080, AM251 + I/R versus I/R). On the contrary, selective CB2R antagonist AM630 had no effect in the infarct size (*P* = 1 versus I/R). Moreover, AM630 (*P* < 0.001, AM630 + propofol + I/R versus propofol + I/R; *P* = 1, AM630 + propofol + I/R versus I/R) but not AM251 (*P* = 1, AM251 + propofol + I/R versus propofol + I/R; *P* < 0.001, AM251 + propofol + I/R versus I/R, (Figures 6(a) and 6(b))).

cTnI is an early phase marker of cardiac ischemia injury.  Figure 6(c) shows that propofol conditioning tended to reduce serum cTnI concentrations compared with I/R (*P* = 0.076) while AM630 tended to increase serum cTnI (*P* = 0.099). AM251 alone had no effect on cTnI concentrations (*P* = 1). When AM630 was pretreated before propofol, the potential effects of propofol in reducing cTnI were completely reversed (*P* = 1, AM630 + propofol + I/R versus propofol + I/R). Of note, AM251 combined with propofol conditioning resulted in significant reduction of cTnI release (*P* = 0.004, AM251 + propofol + I/R versus I/R).

**Figure 7:**
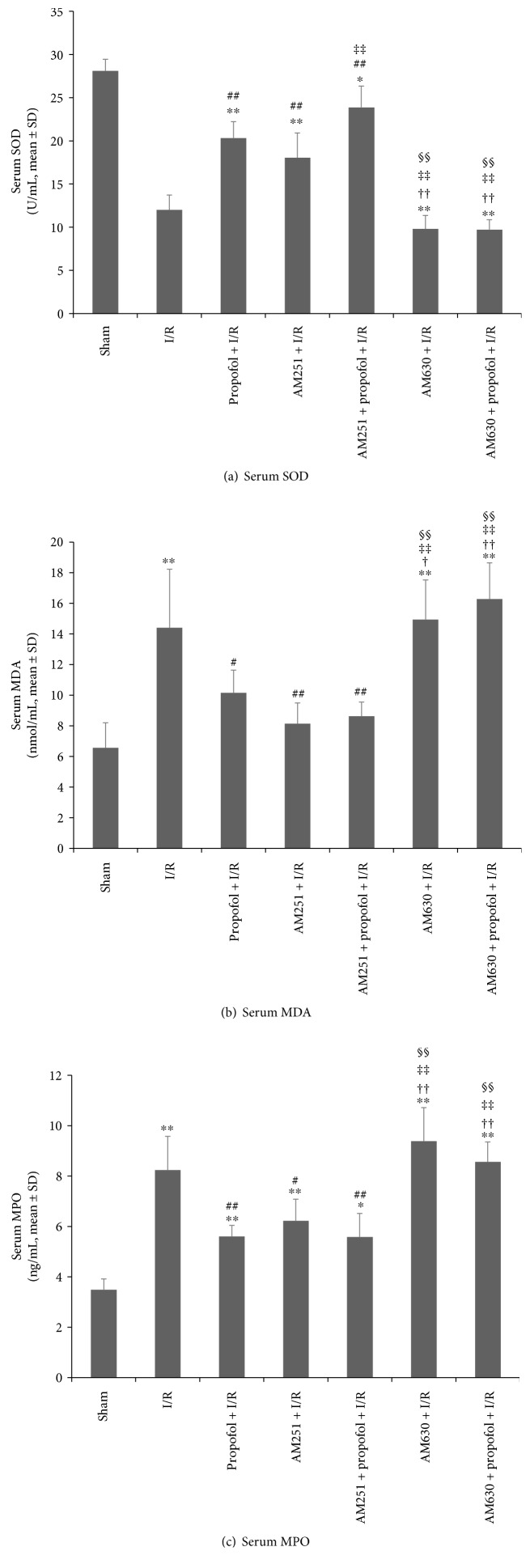
Effects of CB1R and CB2R signaling on propofol conditioning induced antioxidation in vivo. Rat myocardial ischemia/reperfusion (I/R) injury model was set up by ligating left descending coronary artery for 30 minutes and loosening for 24-hour reperfusion. Before and during ischemia, propofol was continuously infused. Selective CB1R antagonist AM251 (1 mg/kg) or selective CB2R antagonist AM630 (1 mg/kg) was injected intravenously 30 minutes before propofol exposure. Serum SOD activities (a), MDA concentrations (b), and MPO concentrations (c) were detected 24 hours after 24-hour reperfusion. The results showed that propofol conditioning was cardioprotective through decreasing oxidation injury. The antioxidative effects of propofol were fully reversed by pretreatment with AM630. *N* = 6 per group. ∗: *P* < 0.05; ∗∗: *P* < 0.01 versus sham. #: *P* < 0.05; ##: *P* < 0.01 versus I/R. ††: *P* < 0.01 versus propofol + I/R. ‡‡: *P* < 0.01 versus AM251 + I/R. §§: *P* < 0.01 versus AM251 + propofol + I/R.

Oxidative redox signaling changes in propofol-induced protection against myocardial I/R were also observed. The results showed that propofol conditioning (*P* < 0.001), AM251 alone (*P* < 0.001), and AM251 combined with propofol conditioning (*P* < 0.001) increased serum SOD concentrations compared with I/R. AM630 injection decreased peripheral SOD activity when compared with sham (*P* < 0.001) and AM630 preexposure fully reversed the effects of propofol conditioning on SOD activity (*P* < 0.001 versus propofol + I/R, Figure 7(a)).

We further measured serum MDA (Figure 7(b)) and myeloperoxidase (MPO) concentrations (Figure 7(c)) to further assess the effects propofol conditioning on oxidative stress. I/R induced significant elevations of serum MDA (*P* < 0.001) and MPO (*P* < 0.001) concentrations compared with sham group. Conditioning with propofol reduced serum MDA (*P* = 0.045) and MPO (*P* = 0.001) concentrations compared with I/R. These antioxidant effects of propofol were completely reversed by AM630 pretreatment (both *P* = 1, AM630 + propofol + I/R versus I/R). AM251 alone (*P* < 0.001) and AM251 combined with propofol conditioning (*P* = 0.002) decreased serum MDA concentrations compared with I/R. Similar effects were found for serum MPO concentrations in the AM251 (*P* < 0.001) and AM251 combined with propofol conditioning (*P* < 0.001) group. AM251 combined with propofol conditioning did not have added effects on propofol alone induced decrease in serum MDA and MPO concentrations (both *P* = 1 versus propofol + I/R).

## 4. Discussion

In the present study, we found that (1) rat cardiomyocyte endocannabinoid (AEA and 2-AG) release was increased by hypoxia and propofol conditioning in vitro. These effects were also observed in vivo in the rat myocardial ischemia/reperfusion injury model. (2) Regulating endocannabinoid levels by inhibiting degradation (URB597) or reuptake (VDM11) before propofol conditioning mimicked the cardiomyocyte protective effects and antioxidative effects of propofol. Furthermore, the effects of propofol overlapped with those of URB597 and VDM11 preincubation. (3) CB2R antagonist AM630 but not CB1R antagonist AM251 reversed the antioxidative effects as well as cardioprotection of propofol conditioning in vivo. These findings collectively suggest that endogenous endocannabinoid release and the consequent CB2R signaling activation modulate propofol conditioning-induced cardioprotection through reducing cardiac oxidative stress.

### 4.1. Propofol Increased Cardiac Endocannabinoid Release

In this study, we found a transient increasing effect of propofol on AEA and 2-AG release. These enhanced effects of release were only observed during propofol exposure and shortly after ischemia (Figure 1) or hypoxia (Figure 2). This time-dependent increase of endocannabinoid by propofol was similar to the results of Patel et al. [19], who reported that intraperitoneal injection of single dose of 100 mg/kg of propofol increased mice whole-brain AEA and 2-AG levels 8 minutes after injection. The concentrations returned to normal 40 minutes later when the mice recovered from anesthesia [19]. In the human study, Schelling et al. found that sevoflurane anesthesia decreased blood AEA concentrations while propofol maintenance was accompanied with stable or higher concentrations of blood AEA [37]. However, in the recent study by Jarzimski et al., propofol anesthesia resulted in similar decreases in serum AEA concentrations to sevoflurane/thiopental anesthesia [22]. The decrease of AEA level was transient and not observed 60 minutes later. In the meantime, serum 2-AG concentration was stable at all time points [22]. On the other hand, although not significant, patients receiving propofol anesthesia always seemed to have higher AEA levels than those receiving sevoflurane/thiopental anesthesia which implied a weak effect of propofol on AEA release [22]. Altogether, these results supported that propofol could increase endocannabinoids, especially AEA release in a short-time period with a small magnitude.

The underlying mechanisms of propofol-induced elevation of AEA and 2-AG release remain to be explored. Mouse brain FAAH has long been found to be inhibited by propofol [19], and our in vitro study also found that propofol (resolved in intralipid) was able to inhibit FAAH (Figure 3(d)). However, contrary results were also reported [22]. Using the human plasma as a source of FAAH, Jarzimski et al. found no change of enzymatic kinetics of FAAH by 50 *μ*M of propofol [22]. These discrepancies suggested a species or organ-specific response of FAAH activity to the presence of propofol. Moreover, endocannabinoids turnover might also be a target of propofol [22]. We did not perform the FAAH activity assay using the rat cardiomyocyte membranes and could not reach the conclusion that propofol increased endocannabinoid release through modulating FAAH activity.

### 4.2. Hypoxia and Ischemia Increased Cardiac Endocannabinoid Release

We found that after hypoxia, endocannabinoid concentrations in the cell culture media experienced a short increase. The duration of such increase seldom lasted for more than 2 hours (Figure 2). The in vivo experiment also found that ischemia resulted in an increase in serum AEA and 2-AG concentrations. The concentrations reached a peak when reperfusion was initiated for 1 hour and returned to normal 4 hours later (Figure 1). These results were similar to previous studies. Wagner et al. found that after 30 minute ischemia and 2-hour reperfusion, AEA and 2-AG contents in the injured myocardium returned to normal [18]. Holman et al. reported a decrease of cardiac 2-AG levels 24 hours after stress and an increase in 2-AG concentrations 2 weeks later [38]. These results encouraged us to speculate that acute stress like ischemia might cause a rapid and transient release of cardiac endocannabinoids which was back to normal several hours later. If the stress continues, a persistent elevation of endocannabinoid concentrations should be observed. In support of this hypothesis, higher concentrations of peripheral blood 2-AG and AEA levels were observed in patients with heart failure [39].

The transient increase of endocannabinoid release by propofol or by myocardial ischemia alone might be beneficial. When FAAH was knocked out and endocannabinoid contents were increased in the ventricle, the mice experienced alleviated age-related cardiac injury [40]. Moreover, a case-control study found a role for allele A of the FAAH 385 variant as a risk factor for myocardial infarction [41]. Our study found that the selective FAAH inhibitor URB597 and reuptake inhibitor VDM11 were cardiomyocyte protective against H/R (Figure 4) which also supported this notion. In the meantime, we could not exclude the possibility that propofol or ischemia might directly act on downstreaming receptor expression, activation, or signaling transduction to exert cardioprotection. Our study on receptor expression also found that propofol conditioning could enhance CB1R and CB2R transcription and expression (Figure 3).

One limitation was that we did not measure cardiac tissue endocannabinoid levels and could not exclude the possibility that increased serum AEA and 2-AG might mainly come from blood. For instance, Wagner et al. found that cardiac ischemia/reperfusion caused an increase of AEA and 2-AG contents in monocytes and platelets [42]. On the other hand, cardiac tissue endocannabinoid levels might not fully reflect cardiac tissue endocannabinoid concentration changes since these endocannabinoids were high degradable with short half-life. Future study that simultaneously measures myocardium and serum endocannabinoid concentrations would help clarify the origins of elevated serum concentrations of AEA and 2-AG.

### 4.3. Propofol Regulated Cardiac Oxidative Redox Signaling

Oxidative stress contributed greatly to cardiomyocyte death due to I/R injury [43]. ROS scavenger has been shown to be able to reduce H/R-induced cardiomyocyte injury [44]. Our experiment found that propofol conditioning could reduce cell ROS production (Figure 5(a)), decrease cardiomyocyte and serum MDA concentrations, (Figures 5(a) and 7(b)) increase serum SOD activity (Figure 7(a)), indicating that propofol could mitigate overoxidation due to reoxygenation. These results were similar to other studies. Propofol was reported to inhibit ischemia-reperfusion injury-induced oxidative stress both in experimental [45] and in clinical settings [46, 47]. Shao et al. found that propofol could dose dependently inhibit the ROS attack from I/R injury [48]. To conclude, propofol conditioning was cardioprotective through regulating cardiac oxidative redox signaling.

In this study, we measured the peripheral blood oxidative stress indicators instead of those at cardiac tissue. Although the peripheral blood indicators were frequently used to reflect oxidation status in cardiac tissue, there might be substantial difference sometimes. Studies have found that autocrine or paracrine mechanisms also played a role in the pathophysiology of myocardial ischemia/reperfusion injury [49]. Future study that simultaneously measures both serum and tissue oxidation status would be helpful.

### 4.4. CB2R Receptor Signaling Meditated Propofol-Induced Cardioprotection

Both CB1R and CB2R were expressed in adult rat cardiomyocytes [50]. In the myocardial I/R model, we found that propofol conditioning-induced antioxidation and cardioprotection could be fully reversed by CB2R antagonist AM630 (Figures 6 and 7). Defer et al. observed the roles of CB2R signaling in oxidative stress-induced cell injury. They found that selective CB2R agonist JWH133 could inhibit apoptosis while AM630 could reverse this effect. In the mouse cardiac I/R model, pretreatment with dual CB1R and CB2R agonists WIN 55212-2 could reduce infarct size through mitigating inflammatory infiltration [51]. Moreover, the effects of WIN 55212-2 could be reversed by AM630 but not AM251, indicating an essential role of CB2R activation in cardioprotection [52]. Using the isolated rat hearts, Lepicier et al. found that both CB1R and CB2R activation could reduce infarct size and the effects of CB1R agonist rely on nitric oxide (NO) production [53]. Moreover, CB1R was found to be more densely expressed in endothelial cells and CB2R primarily expressed in cardiomyocytes [53] and activation of cannabinoid-1 receptor by AEA and HU210 significantly promoted reactive oxygen species-dependent and reactive oxygen species-independent mitogen-activated protein kinase activation and cell death in human coronary artery endothelial cells [54]. These results suggested that both CB1R and CB2R played a role in the pathophysiology of cardiac I/R injury. The further use of different selective CB1R antagonists or gene knock-down models might be helpful to elucidate their roles. Furthermore, whether the NO signaling system played an essential role in propofol-induced cardioprotection and which receptor was responsible remained to be elucidated.

### 4.5. Caveats and Limitations

There could be several limitations or caveats in our study that should be paid attention to. Firstly, the H/R model we used comprised 12-hour hypoxia and 4-hour reoxygenation. The long-term hypoxia might make the cells adaptive to the environment, thereby a dynamic change in endocannabinoid signaling might exist. Future studies were needed to clarify it. Secondly, the effects of endocannabinoid release on cardioprotection was only observed in vitro, but not in in vivo study. However, considering the fact that cardiac endocannabinoid signaling in vivo could be influenced by cardiomyocytes, other cardiac cells, circulation, and neuroendocrine states [55, 56], the results that could gained in in vivo results might be different from the findings in our in vitro study. Nevertheless, the findings gained in our in vitro study identified the downstream signaling pathways in association with CB2R activation and cardioprotection of propofol conditioning that may foster further mechanistic studies. Thirdly, we have identified increases in CB1R and CB2R protein and mRNA expression by propofol. Whether the one-third increase in protein contents or 3- to 5-fold increase in mRNA levels was biologically effective was not known. Lin et al. found that chronic kidney disease (CKD) could increase cardiac CB1R protein expression by more than 50%, which they thought was biologically significant [57]. In patients with severe heart failure, CB1R RNA was downregulated 0.7-fold, whereas CB2R was upregulated more than 11-fold which was accompanied with significantly elevated peripheral blood levels of endocannabinoids [39]. Based on these results, there was a possibility that the increased CB1R and CB2R expression induced by propofol might not play a significant role in biological processes. Another limitation needed to mention is that we did not utilize CB1R and CB2R antagonists to clarify whether these receptors signaling or other nonspecific receptors were responsible for propofol-induced preservation of cell viability and reduction of oxidative stress. Moreover, these experiments could help us differentiate the roles of endocannabinoids and receptors in cardioprotection by propofol considering that propofol could induce both endocannabinoid release and receptor expression.

In conclusion, propofol conditioning showed protection against cardiomyocyte hypoxia and myocardial ischemia. The protection was accompanied with and dependent on increased endocannabinoid (AEA and 2-AG) release and following alleviation of oxidative stress. CB2R signaling but not CB1R signaling activation meditated propofol conditioning-induced cardioprotection and antioxidation.
